# Locating the propagation source in complex networks with observers-based similarity measures and direction-induced search

**DOI:** 10.1007/s00500-023-08000-7

**Published:** 2023-04-04

**Authors:** Fan Yang, Chungui Li, Yong Peng, Jingxian Liu, Yabing Yao, Jiayan Wen, Shuhong Yang

**Affiliations:** 1 School of Computer Science and Technology, Guangxi University of Science and Technology, Liuzhou, 545006 China; 2 School of Computer and Communication, Lanzhou University of Technology, Lanzhou, 730050 China; 3 Key Laboratory of Intelligent Information Processing and Graph Processing, Guangxi University of Science and Technology, Liuzhou, 545006 China

**Keywords:** Complex networks, Propagation source locating, Direction-induced search (DIS), Observers-based similarity measures, Diffusion direction information, Infection time information

## Abstract

Locating the propagation source is one of the most important strategies to control the harmful diffusion process on complex networks. Most existing methods only consider the infection time information of the observers, but the diffusion direction information of the observers is ignored, which is helpful to locate the source. In this paper, we consider both of the diffusion direction information and the infection time information to locate the source. We introduce a relaxed direction-induced search (DIS) to utilize the diffusion direction information of the observers to approximate the actual diffusion tree on a network. Based on the relaxed DIS, we further utilize the infection time information of the observers to define two kinds of observers-based similarity measures, including the Infection Time Similarity and the Infection Time Order Similarity. With the two kinds of similarity measures and the relaxed DIS, a novel source locating method is proposed. We validate the performance of the proposed method on a series of synthetic and real networks. The experimental results show that the proposed method is feasible and effective in accurately locating the propagation source.

## Introduction

In the modern world, the ubiquity of the diffusion phenomena taking on networks has incurred huge losses to human society. Some typical examples include computer virus propagation (Wang et al. 2014a), disease spreading (Zhang et al. 2018) and rumor diffusion (Hosseini and Azgomi 2016), etc. It is of great theoretical and practical significance to develop effective strategies to control the harmful diffusion process (Yu et al. 2017). As one of the significant measures, propagation source locating has attracted widespread attentions, many effective methods are proposed in recent years (Jiang et al. 2017; Paluch et al. 2020). These methods can provide effective solutions for many important issues in reality, including locating the source(s) of SARS (Brockmann and Helbing 2013), COVID-19 (Tian et al. 2020), Cholera (Li et al. 2021), identifying the source of delay in public transportation networks (Manitz et al. 2017), estimating the source of foodborne disease (Horn and Friedrich 2019), etc.

It is well known that, when a diffusion process occurs on a network, there exists a spanning tree corresponding to the first time each node gets infected (Shah and Zaman 2011; Pinto et al. 2012; Tang et al. 2018). In fact, reconstructing the spanning tree is helpful to locate the propagation source (Yang et al. 2020). However, the commonly used breadth-first search (BFS) heuristic (Shah and Zaman 2011; Pinto et al. 2012; Yang et al. 2016) may be not an effective strategy (Tang et al. 2018; Yang et al. 2020). In this paper, we introduce an effective graph traversal method termed as relaxed direction-induced search (DIS), which is developed in our previous work (Yang et al. 2020). By utilizing the diffusion direction information of the observers, the relaxed DIS could effectively approximate the spanning tree corresponding to the first time each node gets infected. Based on the relaxed DIS, we further utilize the infection time information of the observers to define two kinds of observers-based similarity measures: (1) Infection Time Similarity, which measures the similarity between the observation infection time of the given observers and the measuring infection time of the given observers. (2) Infection Time Order Similarity, which measures the similarity between two sorted sequences of the given observers. One sequence is the observers ascending order obtained by sorting the observation infection time of the observers. Another sequence is the observers ascending order obtained by sorting the measuring infection time of the observers. Further, with the two kinds of similarity measures and the relaxed DIS, we propose a novel source locating method. Obviously, in the proposed method, both of the diffusion direction information and the infection time information are considered. Experiments are performed on a series of synthetic and real networks; the results show that the proposed method is feasible and effective in accurately locating the propagation source.

**Fig. 1:**
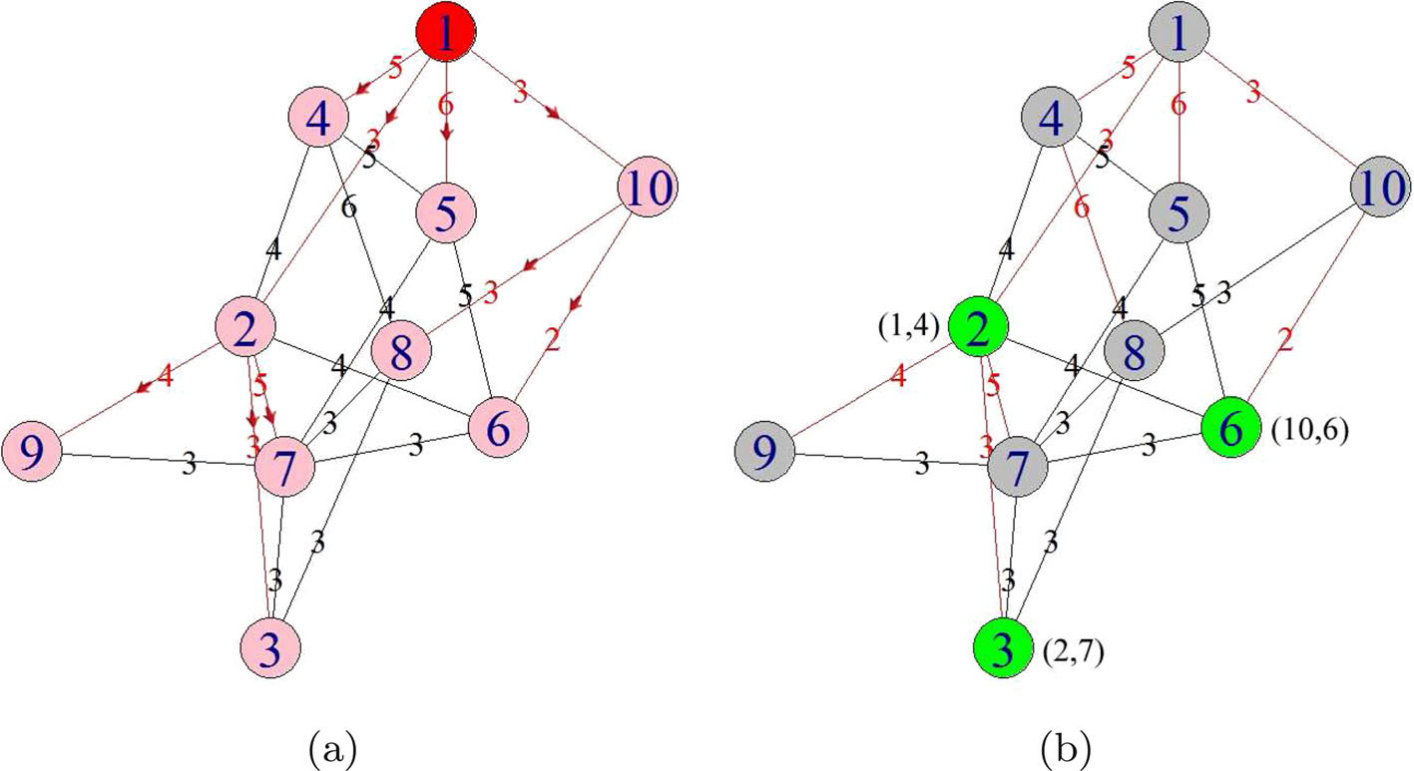
Sub-Fig. 1a shows the SI model ($\beta =1$) diffused on a given network. The infection is initiated by node 1 (with “red” color). All the “red” paths form a diffusion tree (rooted at node 1) of the network. The arrows attached to the “red” paths represent the actual diffusion direction. The infection diffused along this actual diffusion tree. The nodes with “pink” color are in the infectious state. Sub-Fig. 1b shows a relaxed DIS spanning tree (rooted at node 1) of the network in Sub-Fig. 1a. This tree is constructed by the edges with “red” color, which is generated by the relaxed DIS algorithm with three observers (nodes 2, 3 and 6, with “green” color). The nodes with “gray” color cannot be observed. The pair of value next to each observer represents the recorded Diffusion Direction information and Diffusion Timing information (color figure online)

**Table 1:** Notation summarization

Notation	Definition
$\mathcal {G}$	The topological graph of network
$\mathcal {V}$	The nodes set in $\mathcal {G}$
$\mathcal {E}$	The edges set in $\mathcal {G}$
$\varvec{\theta }$	The propagation delay set associated with $\mathcal {E}$
$\mathcal {O}$	Observers set
*o*	Observer
$\mathcal {K}$	The number of observers
$s^*$	The propagation source
$\beta$	The propagation ratio of SI model
$\vert \cdot \vert$	Calculating the number of element

The rest of this paper is organized as follows. Existing related works are briefly reviewed in Sect. 2. We introduce the direction-induced search (DIS) in Sect. 3. Our method is proposed in Sect. 4. The performance of the proposed method is validated in Sect. 5. We conclude this work in Sect. 6.

## Related work

For unweighted networks, a systematic method for propagation source locating was pioneered by Shah et al. (2011); they constructed a source estimator based on a novel topological quantity which is termed as Rumor Centrality (RC). Some researchers extended the RC to more complex environments, such as utilizing multiple observations to locate the source (Wang et al. 2014b), locating multi-sources (Luo et al. 2013; Wang et al. 2015) and so on. Zhu et al. (2016) developed a sample path-based method termed as Jordan Center (JC). Several improved methods based on the JC were developed to locate the source(s) with sparse observations (Zhu and Ying 2014; W.Luo et al. 2014; Jiang et al. 2018). Meanwhile, many source locating methods based on various ideas were developed for unweighted networks, including the Dynamic Message Passing-based method (Lokhov et al. 2014), the Belief Propagation base method (Altarelli et al. 2014), the Minimum Description Length-based method (Prakash et al. 2014), the Monte Carlo-based method (Antulov-Fantulin et al. 2015), the Rationality Observation-based method (Yang et al. 2016), the Time Aggregated Graph-based method (Chai et al. 2021), etc. The above methods are effective in unweighted networks. However, in reality, we have to consider various significant weights associated with the edges in networks, such as the traffic, the propagation delay and so on.

For weighted networks, Brockmann et al. (2013) modeled the Global Mobility Network as a weighted graph and proposed a source locating method based on a novel effective distance. This method is extended to more complex environments, including identifying the multiple sources (Jiang et al. 2015), identifying the source of delay in public transportation networks (Manitz et al. 2017), etc. However, the effective distance-based methods require the complete knowledge of nodes state. Meanwhile, there are several source locating methods based on various ideas for weighted networks  (Cai et al. 2018; Chang et al. 2020; Feizi et al. 2019). But these methods also require the complete knowledge of nodes state. In reality, it is often the case that only limited nodes state can be observed (Caputo et al. 2019). To this problem, many methods were developed to locate the source with limited observers. Shen et al. (2016) developed a time-reversal backward spreading (TRBS) algorithm, but this algorithm may not work if the locatability condition is violated. Hu et al. (2019) proposed a greedy optimization algorithm to reduce the number of observers for TRBS. Tang and Ji et al. (2018) et al. proposed a source estimation algorithm based on the Gromov matrix. However, the Gromov matrix may be not the optimal heuristic for source locating. Meanwhile, Fu et al. (2016) proposed a backward diffusion-based method for multiple sources locating. Wang (2019) and Xu et al. (2019) identified the diffusion source based on the Spearman’s coefficient. Wang and Sun (2020) proposed a sequential neighbor filtering (SNF) algorithm for heterogeneous propagation models. Wang et al. (2021) proposed three source locating algorithms by defining the estimated mean and standard deviation of the propagation delay. However, the methods using limited observers only considered the infection time information, but the diffusion direction information was ignored.

**Table 2:** The time complexity of Gauss, GSSI, TRBS, SNF, OSBFS and OSDIS algorithms

Algorithm	Time complexity
Gauss	$O\left( \vert \mathcal {V}\vert ^3\right)$ (Pinto et al. 2012)
GSSI	$O\left( \vert \mathcal {V}\vert ^3+\vert \mathcal {V}\vert \mathcal {K}^3\right)$ (Tang et al. 2018)
TRBS	$O\left( \mathcal {K}N\log N\right)$ (Shen et al. 2016)
SNF	$O\left( \vert \mathcal {V}\vert ^2\vert D\vert \right)$ (Wang et al. 2022)
OSBFS	$O\left( \vert \mathcal {V}\vert ^3+z\vert \mathcal {V}\vert ^2\mathcal {K}\right)$
OSDIS	$O\left( \vert \mathcal {V}\vert ^3+z\vert \mathcal {V}\vert ^2\mathcal {K}\right)$

**Table 3:** The parameters for generating the synthetic networks

Networks	Parameters
BA model (1)	barabasi.game (200, power=1.5, m=2, directed=F)
BA model (2)	barabasi.game (200, power=1.7, m=2, directed=F)
BA model (3)	barabasi.game (200, power=1.9, m=2, directed=F)
BA model (4)	barabasi.game (200, power=2.1, m=2, directed=F)
BA model (5)	barabasi.game (200, power=2.3, m=2, directed=F)
BA model (6)	barabasi.game (200, power=2.5, m=2, directed=F)
WS model (1)	watts.strogatz.game (1, 200, 2, 0.2)
WS model (2)	watts.strogatz.game (1, 200, 2, 0.4)
WS model (3)	watts.strogatz.game (1, 200, 2, 0.6)
WS model (4)	watts.strogatz.game (1, 200, 2, 0.8)
WS model (5)	watts.strogatz.game (1, 200, 2, 1.0)

Software environment: R 64 $\times$ 3.3.3 + igraph R 1.2.1

The Gaussian estimator (Pinto et al. 2012) first located the source with limited observers by utilizing the diffusion direction information of the observers, and its time complexity can be reduced by ignoring the observers with low-quality information (Paluch et al. 2018). However, the diffusion direction information is only used in the tree graphs. In our previous work (Yang et al. 2020), a relaxed direction-induced search (DIS) was proposed by utilizing the diffusion direction information. With the relaxed DIS, the accuracy of the Gaussian estimator on general graphs is improved. Different from the previous work, in this paper, we first introduce the relaxed direction-induced search (DIS) (Yang et al. 2020) to utilize the diffusion direction information of the observers to approximate the actual diffusion tree on a network. Based on the relaxed DIS, we further utilize the infection time information of the observers to define two kinds of similarity measures, including the Infection Time Similarity and the Infection Time Order Similarity. With the two kinds of similarity measures and the relaxed DIS, we propose a novel source locating method. Obviously, the diffusion direction information and the infection time information are combined in this method. The feasibility and effectiveness of this method are validated on a series of synthetic and real networks.

**Table 4:** Real networks

Networks	Data source
Dolphins	http://networkrepository.com/dolphins.php
Lesmis	http://networkrepository.com/lesmis.php
PDZBase(LCC)	http://konect.cc/networks/maayan-pdzbase/
USAirlines	http://networkrepository.com/USAir97.php
NetScience(LCC)	http://konect.cc/networks/dimacs10-netscience/
Celegans	http://konect.cc/networks/dimacs10-celegans_metabolic/
Euroroads(LCC)	http://konect.cc/networks/subelj_euroroad/

*LCC* the abbreviation of largest connected component

**Table 5:** The topological properties of the used networks

Network	$\vert \mathcal {V}\vert$	$\vert \mathcal {E}\vert$	$\left\langle k\right\rangle$	*A*	APL
BA model (1)	200	397	3.97	$-$0.321	2.85
BA model (2)	200	397	3.97	$-$0.532	2.43
BA model (3)	200	397	3.97	$-$0.501	2.13
BA model (4)	200	397	3.97	$-$0.807	2.04
BA model (5)	200	397	3.97	$-$0.757	2.02
BA model (6)	200	397	3.97	$-$0.806	2.00
WS model (1)	200	400	4.00	0.102	4.39
WS model (2)	200	400	4.00	0.0003	4.04
WS model (3)	200	400	4.00	0.044	4.03
WS model (4)	200	400	4.00	$-$0.059	3.96
WS model (5)	200	400	4.00	$-$0.051	3.91
Dolphins	62	159	5.13	$-$0.044	3.36
Lesmis	77	254	6.60	$-$0.165	2.64
PDZBase	161	209	2.60	$-$0.466	5.33
USAirlines	332	2126	12.81	$-$0.208	2.74
Netscience	379	914	4.82	$-$0.082	6.04
Celegans	453	2025	8.94	$-$0.226	2.66
Euroroads	1039	1305	2.51	0.090	18.40

$\left\langle k\right\rangle$ the average degree of $\mathcal {G}$

*A* the assortative coefficient (Newman 2002)

*APL* the average path length (the number of edges)

## Preliminaries

A network is modeled as an undirected and weighted graph $\mathcal {G}=\left( \mathcal {V}, \mathcal {E}, {\varvec{\theta }}\right)$, where $\mathcal {V}$ and $\mathcal {E}$ represent the nodes set and edges set, respectively. ${\varvec{\theta }}=\left\{ \theta _{uv}\right\}$, where $\theta _{uv}$ denotes the random propagation delay associated with an edge connecting nodes *u* and *v*, $u, v\in \mathcal {V}$, $vu\in \mathcal {E}$. The random variables $\theta _{vu}$ for different edges *vu* have a known, arbitrary joint distribution.

*Diffusion model* Similar to the references (Zhu and Ying 2016; W.Luo et al. 2014; Lokhov et al. 2014; Yang et al. 2016), the diffusion process on $\mathcal {G}$ is discrete. We adopt a simple Susceptible-Infectious (SI) model. With the SI model, each node in ${\mathcal {V}}$ is only in one of the two states: (1) susceptible, if it has not been infected so far, or (2) infectious, if it has been infected by any one neighbor. The diffusion process on $\mathcal {G}$ is initiated by a single propagation source (denoted by $s^*$) at an unknown time $t^*$. All nodes are susceptible except for $s^*$ is infectious. A diffusion is possible from an infected node to a susceptible node if and only if there is an edge between them. Once infected, the node will stay the infectious state forever. Let $\mathcal {N}\left( v\right)$ denote the neighbors set of node *v*, suppose *v* is infected by one neighbor *w* at time $t_{v}$, then *v* will attempt to infect each susceptible neighbor $u\in \mathcal {N}\left( v\right)$ (except for *w*) along the weighted edge *vu* with propagation ratio $\beta$. If there are two or more infected neighbors having a same propagation delay to *u*, *u* can be first time infected by only one neighbor. Without loss of generality, the diffusion process is terminated when there are no susceptible nodes in $\mathcal {G}$.

**Fig. 2:**
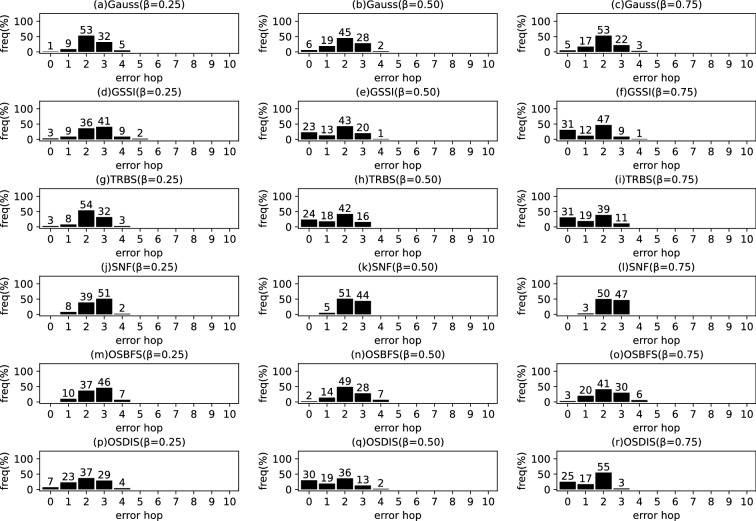
The results of Gauss, GSSI, TRBS, SNF, OSBFS and OSDIS algorithms applied on BA model (1). Each sub-figure is obtained by 100 runs

**Fig. 3:**
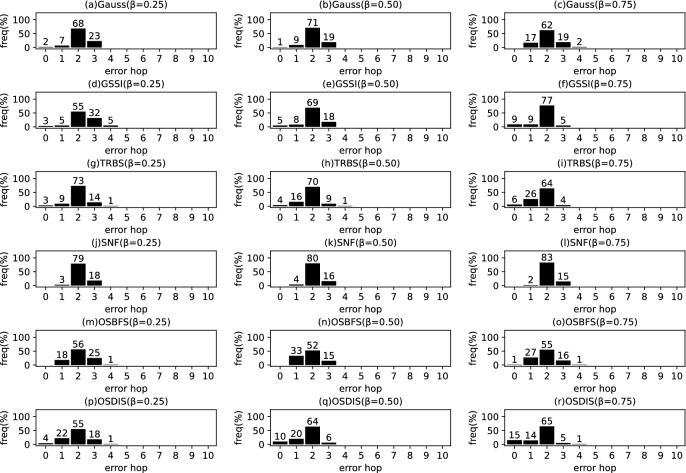
The results of Gauss, GSSI, TRBS, SNF, OSBFS and OSDIS algorithms applied on BA model (2). Each sub-figure is obtained by 100 runs

**Fig. 4:**
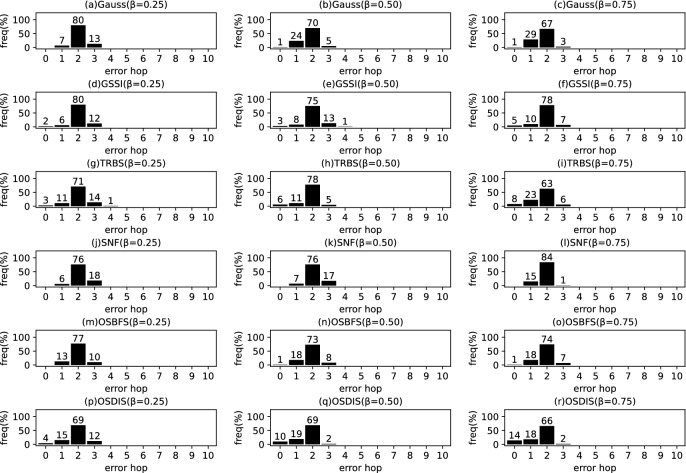
The results of Gauss, GSSI, TRBS, SNF, OSBFS and OSDIS algorithms applied on BA model (3). Each sub-figure is obtained by 100 runs

**Fig. 5:**
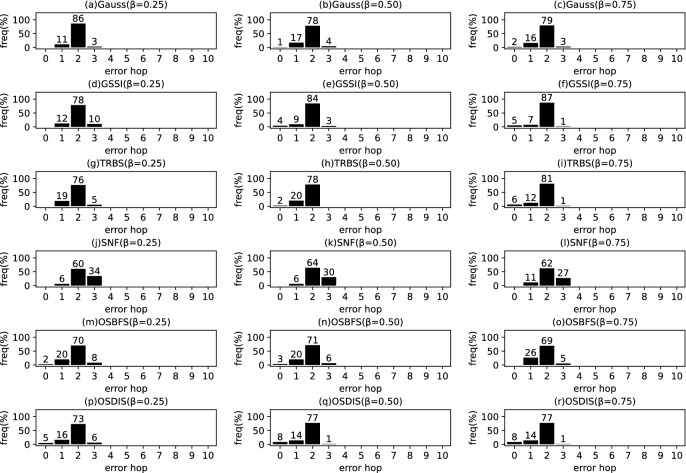
The results of Gauss, GSSI, TRBS, SNF, OSBFS and OSDIS algorithms applied on BA model (4). Each sub-figure is obtained by 100 runs

**Fig. 6:**
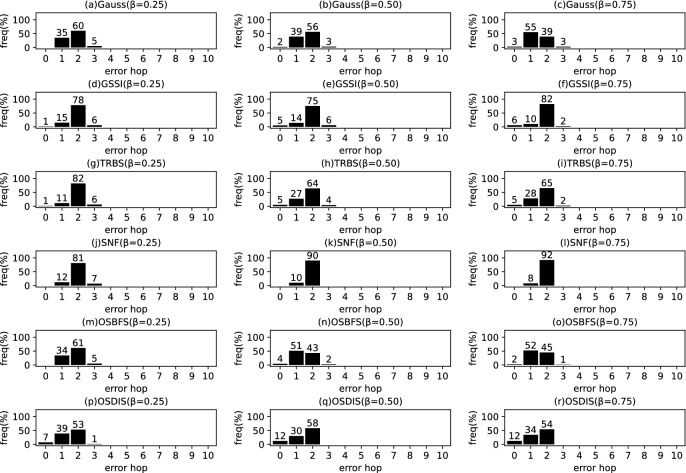
The results of Gauss, GSSI, TRBS, SNF, OSBFS and OSDIS algorithms applied on BA model (5). Each sub-figure is obtained by 100 runs

**Fig. 7:**
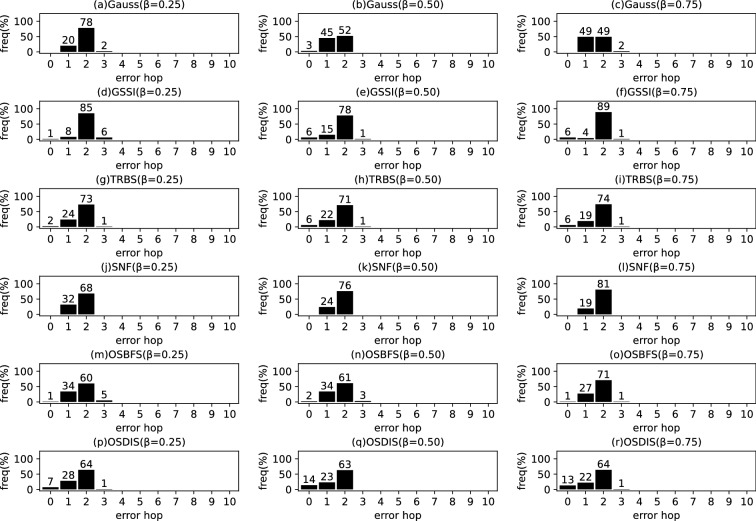
The results of Gauss, GSSI, TRBS, SNF, OSBFS and OSDIS algorithms applied on BA model (6). Each sub-figure is obtained by 100 runs

Let $\mathcal {O}=\left\{ o_k\right\} ^{\mathcal {K}}_{k=1}\subseteq \mathcal {V}$ denote the set of $\mathcal {K}$ observable nodes on $\mathcal {G}$, termed as observers set, whose location in $\mathcal {G}$ is known. Generally, there is $\mathcal {K}\ll \vert \mathcal {V}\vert$. Similar to the references (Pinto et al. 2012; Yang et al. 2020), each $o_k\in \mathcal {O}$ can provide two types of information: (1) the Diffusion Direction information in which the infection arrives to $o_k$, (2) the Infection Timing information at which the infection arrives to $o_k$.
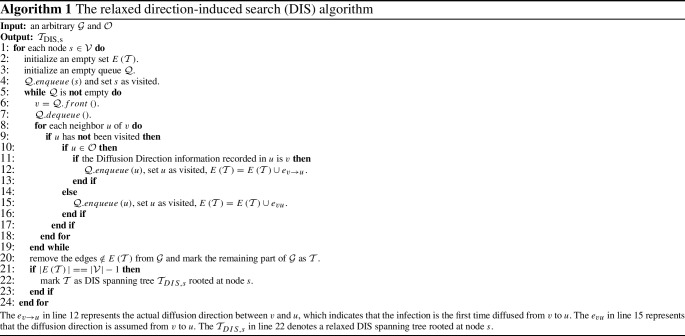


*Direction-induced search (DIS)* We introduce a graph traversal method termed as relaxed direction-induced search (DIS), which is developed in our previous work (Yang et al. 2020). The relaxed DIS is summarized in Algorithm 1. The $E\left( \mathcal {T}\right)$ declared in line 2 is an edge set. The function of lines 3–19 is to traverse the $\mathcal {G}$ with *s* as root, which requires $O\left( \vert \mathcal {V}\vert ^2\right)$ computations in the worst case. Here, from lines 10–16, we know that, if a node is an observer, then the infection direction is determined by the Diffusion Direction information recorded in this node. If the node is a non-observer, the infection direction will be assumed to be its current parent node. From lines 20–23, a DIS spanning tree ${\mathcal {T}}_{\textrm{dis},s}$ is generated if and only if “$\vert E\left( \mathcal {T} \right) \vert ==\vert V\vert -1$”, where line 20 requires $O\left( \vert \mathcal {V}\vert +\vert \mathcal {E}\vert \right)$ computations. Finally, taking the loop in line 1 into account, the time complexity of Algorithm 1 is $O\left( \vert \mathcal {V}\vert ^3\right)$. Further, by using Algorithm 1, a relaxed DIS spanning tree is generated by utilizing the Diffusion Direction information recorded in $\mathcal {O}$.

**Table 6:** The average error hop of the six algorithms on different networks

$\beta$	Network	Gauss	GSSI	TRBS	SNF	OSBFS	OSDIS
0.25	BA model (1)	2.31	2.50	2.24	2.47	2.50	**2**.**00**
	BA model (2)	2.12	2.31	2.01	2.15	2.09	**1**.**90**
	BA model (3)	2.06	2.02	1.99	2.12	1.97	**1**.**89**
	BA model (4)	1.92	1.98	1.86	2.28	1.84	**1**.**80**
	BA model (5)	1.70	1.89	1.93	1.95	1.71	**1**.**48**
	BA model (6)	1.82	1.96	1.73	1.68	1.69	**1**.**59**
	WS model (1)	2.61	2.34	2.34	2.93	2.77	**1**.**81**
	WS model (2)	2.60	2.26	2.10	3.08	2.84	**1**.**78**
	WS model (3)	2.69	2.26	2.18	2.99	3.62	**1**.**80**
	WS model (4)	2.61	2.30	2.14	2.89	3.39	**1**.**64**
	WS model (5)	2.32	2.12	1.86	2.79	3.56	**1**.**85**
	Dolphins	2.11	2.58	1.98	2.56	2.76	**1**.**60**
	Lesmis	2.19	2.16	2.08	2.60	2.10	**1**.**70**
	PDZBase	3.07	2.32	2.27	3.42	3.70	**1**.**99**
	USAirlines	2.26	2.19	2.19	2.29	2.73	**1**.**89**
	NetScience	3.30	2.06	2.39	2.74	3.28	**1**.**94**
	Celegans	2.22	2.26	2.11	2.22	2.39	**1**.**84**
	Euroroads	6.28	**3**.**44**	6.08	6.71	12.01	4.47
0.50	BA model(1)	2.01	1.63	1.50	2.39	2.24	**1**.**38**
	BA model (2)	2.08	2.00	1.87	2.12	1.82	**1**.**66**
	BA model (3)	1.79	2.01	1.82	2.10	1.88	**1**.**63**
	BA model (4)	1.85	1.86	1.76	2.24	1.80	**1**.**71**
	BA model (5)	1.60	1.82	1.67	1.90	**1**.**43**	1.46
	BA model (6)	**1**.**49**	1.74	1.67	1.76	1.65	**1**.**49**
	WS model (1)	2.12	1.35	0.98	2.97	1.76	**0**.**88**
	WS model (2)	2.20	1.25	**1**.**01**	2.85	2.78	1.23
	WS model (3)	2.06	1.29	1.21	2.86	3.37	**1**.**17**
	WS model (4)	2.21	1.06	0.97	2.80	3.18	**0**.**95**
	WS model (5)	2.17	1.30	1.35	3.03	3.49	**1**.**10**
	Dolphins	2.21	2.19	1.68	2.74	2.47	**1**.**37**
	Lesmis	2.10	1.51	1.66	2.70	1.77	**1**.**39**
	PDZBase	2.57	1.65	1.71	3.38	3.04	**1**.**47**
	USAirlines	2.21	1.83	1.84	2.06	2.31	**1**.**80**
	NetScience	3.07	1.77	2.03	2.58	2.36	**1**.**55**
	Celegans	1.98	1.78	1.74	2.29	2.24	**1**.**56**
	Euroroads	6.52	2.56	4.22	6.69	8.14	**2**.**53**
0.75	BA model(1)	2.01	1.37	1.50	2.44	2.16	**1**.**36**
	BA model (2)	2.06	1.78	1.66	2.13	1.89	**1**.**63**
	BA model (3)	1.72	1.87	1.67	1.86	1.87	**1**.**56**
	BA model (4)	1.83	1.84	1.77	2.16	1.79	**1**.**71**
	BA model (5)	**1**.**42**	1.80	1.64	1.92	1.45	**1**.**42**
	BA model (6)	**1**.**53**	1.85	1.70	1.81	1.72	**1**.**53**
	WS model (1)	1.82	**0**.**29**	0.36	2.96	1.90	0.66
	WS model (2)	1.90	0.37	**0**.**29**	2.68	2.28	0.79
	WS model (3)	2.22	**0**.**46**	0.51	2.77	3.15	1.03
	WS model (4)	1.86	0.48	**0**.**25**	2.69	3.04	1.00
	WS model (5)	1.80	0.48	**0**.**42**	2.78	3.25	0.85
	Dolphins	1.95	2.00	1.45	2.85	2.13	**1**.**29**
	Lesmis	2.00	1.56	1.27	2.75	1.61	**1**.**22**
	PDZBase	2.68	1.37	1.45	3.34	2.56	**1**.**31**
	USAirlines	2.10	**1**.**24**	1.36	1.98	2.21	1.52
	NetScience	3.02	1.44	1.54	2.38	1.92	**1**.**37**
	Celegans	1.98	1.08	**1**.**05**	1.92	2.27	1.29
	Euroroads	5.89	1.78	2.29	7.12	3.74	**1**.**64**

The values with bold font are the optimal values

Frequently used notations are summarized in Table 1.

## The proposed method

Given an arbitrary $\mathcal {G}$ and an arbitrary $\mathcal {O}$, we locate the propagation source by measuring the similarity between the $\mathcal {O}$ in the actual diffusion tree (corresponding to the first time each node gets infected) and the $\mathcal {O}$ in a spanning tree of $\mathcal {G}$, which can be described as an estimator that maximizes the similarity.

1 $$\begin{aligned} \hat{s}=\mathop {\arg \max }_{s\in \mathcal {V}}\varvec{\mathcal {S}}\left( \mathcal {O}_{\mathcal {T}_{s^*}}, \mathcal {O}_{\mathcal {T}_s}\right) \end{aligned}$$

where $\mathcal {T}_{s^*}$ denotes the actual diffusion tree with source $s^*$ as root, and $\mathcal {T}_s$ denotes a tree that spans all nodes in $\mathcal {G}$ with node *s* as root. $\mathcal {O}_{\mathcal {T}_{s^*}}$ and $\mathcal {O}_{\mathcal {T}_s}$ denote the given $\mathcal {O}$ in $\mathcal {T}_{s^*}$ and $\mathcal {T}_s$, respectively. $\varvec{\mathcal {S}}\left( \mathcal {O}_{\mathcal {T}_{s^*}}, \mathcal {O}_{\mathcal {T}_s}\right)$ denotes the similarity between $\mathcal {O}_{\mathcal {T}_{s^*}}$ and $\mathcal {O}_{\mathcal {T}_s}$.

Theoretically, we have to evaluate $\varvec{\mathcal {S}}\left( \mathcal {O}_{\mathcal {T}_{s^*}}, \mathcal {O}_{\mathcal {T}_s}\right)$ in Eq. 1 for all spanning trees of $\mathcal {G}$ and then select the one with the maximal similarity and its root is the $s^*$. However, the complexity to generate all spanning trees of $\mathcal {G}$ will increase exponentially with the number of nodes. Therefore, we introduce an approximation by assuming that the actual diffusion tree is a relaxed DIS spanning tree (obtained by Algorithm 1), and the time complexity only requires $O\left( \vert \mathcal {V}\vert ^3\right)$. Then, Eq. 1 can be modified as follows.

2 $$\begin{aligned} \hat{s}=\mathop {\arg \max }_{s\in \mathcal {V}}\varvec{\mathcal {S}}\left( \mathcal {O}_{\mathcal {T}_{s^*}}, \mathcal {O}_{\mathcal {T}_{{\text {DIS}},s}}\right) \end{aligned}$$

where $\mathcal {T}_{{\text {DIS}},s}$ is a relaxed DIS spanning tree of $\mathcal {G}$ with a node *s* as root. $\mathcal {O}_{\mathcal {T}_{{\text {DIS}},s}}$ denote the given $\mathcal {O}$ in $\mathcal {T}_{{\text {DIS}},s}$. $\varvec{\mathcal {S}}\left( \mathcal {O}_{\mathcal {T}_{s^*}}, \mathcal {O}_{\mathcal {T}_{{\text {DIS}},s}}\right)$ denotes the similarity between $\mathcal {O}_{\mathcal {T}_{s^*}}$ and $\mathcal {O}_{\mathcal {T}_{{\text {DIS}},s}}$.

Since $\mathcal {K}<\vert \mathcal {V}\vert$, $\mathcal {T}_{{\text {DIS}},s}$ may be not unique, and each $\mathcal {T}_{{\text {DIS}},s}$ may not correspond to the actual diffusion tree. Thus, the relaxed DIS is obviously a sub-optimal heuristic.

### Observers-based similarity measures

In this subsection, we first define two kinds of observers-based similarity measures by utilizing the infection time information of observers. One is Infection Time Similarity; another is Infection Time Order Similarity.

#### Definition 1

Observation Infection Time. Given an arbitrary $\mathcal {G}=\left( \mathcal {V},\mathcal {E},{\varvec{\theta }}\right)$ and an observers set $\mathcal {O}=\left\{ o_k\right\} ^{\mathcal {K}}_{k=1}$. the Observation Infection Time of $\mathcal {O}$ is defined as ${{\textbf {T}}}_{\mathcal {O}}=\left\{ t_{o_k}\right\} ^{\mathcal {K}}_{k=1}$, where $t_{o_k}$ denotes the Infection Timing information recorded in $o_k$.

**Fig. 8:**
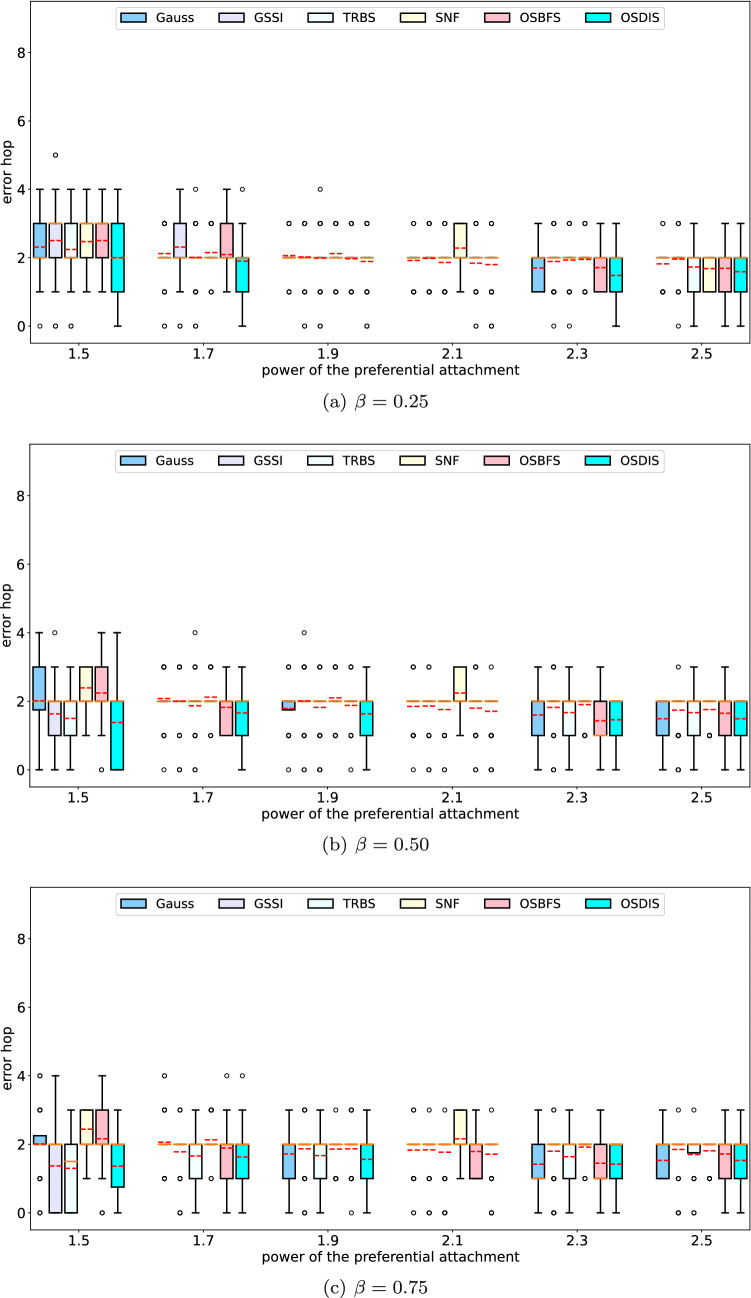
The IQR (box-plot) of error hop of Gauss, GSSI, TRBS, SNF, OSBFS and OSDIS algorithms on the BA models generated by different powers of the preferential attachment. The orange line and red line in each box denote the median and average error hop (also shown in Table 6), respectively (color figure online)

**Table 7:** The average error delay of the six algorithms on different networks

$\beta$	Network	Gauss	GSSI	TRBS	SNF	OSBFS	OSDIS
0.25	BA model (1)	8.56	9.07	8.29	8.94	9.56	**7**.**03**
	BA model (2)	7.89	8.33	7.52	8.13	7.92	**6**.**95**
	BA model (3)	7.68	8.01	7.59	8.39	7.63	**7**.**20**
	BA model (4)	6.95	7.64	6.87	9.21	7.00	**6**.**51**
	BA model (5)	5.98	7.11	7.14	7.53	6.54	**5**.**33**
	BA model (6)	6.66	7.54	6.36	6.64	6.56	**5**.**73**
	WS model (1)	9.61	8.80	8.57	11.34	10.40	**6**.**73**
	WS model (2)	10.04	8.54	7.79	11.87	10.99	**6**.**53**
	WS model (3)	10.09	8.57	8.11	11.37	13.23	**6**.**48**
	WS model (4)	9.74	8.72	8.11	11.71	13.02	**6**.**24**
	WS model (5)	8.75	7.89	7.04	10.92	13.49	**6**.**81**
	Dolphins	7.68	9.53	7.39	9.13	9.60	**5**.**79**
	Lesmis	8.22	7.82	7.53	9.68	7.86	**6**.**36**
	PDZBase	12.07	8.94	8.90	13.60	14.24	**7**.**54**
	USAirlines	8.01	7.82	7.70	8.09	9.95	**6**.**74**
	NetScience	13.00	7.98	9.31	11.33	12.82	**7**.**62**
	Celegans	7.70	7.77	7.29	7.89	8.36	**6**.**40**
	Euroroads	24.93	**13**.**59**	24.48	27.00	47.75	17.93
0.50	BA model(1)	7.60	6.01	5.60	9.07	8.49	**4**.**88**
	BA model (2)	7.51	7.64	7.03	8.05	6.87	**5**.**92**
	BA model (3)	6.71	7.43	6.93	8.31	7.10	**6**.**05**
	BA model (4)	7.20	6.90	6.47	9.04	6.92	**6**.**05**
	BA model (5)	5.75	6.49	5.93	7.13	5.32	**5**.**00**
	BA model (6)	5.41	6.28	5.78	6.98	6.24	**5**.**20**
	WS model (1)	8.02	4.89	3.57	11.83	6.27	**3**.**16**
	WS model (2)	8.17	4.69	**3**.**87**	10.89	10.48	4.52
	WS model (3)	7.21	4.64	4.38	10.70	12.61	**4**.**26**
	WS model (4)	8.32	4.05	3.82	11.39	12.29	**3**.**59**
	WS model (5)	8.06	4.97	5.02	11.63	12.94	**3**.**97**
	Dolphins	7.97	8.16	6.18	9.95	8.79	**4**.**92**
	Lesmis	7.73	5.48	6.14	10.57	6.70	**5**.**39**
	PDZBase	10.16	6.24	6.50	13.21	11.61	**5**.**54**
	USAirlines	7.55	**6**.**35**	6.52	7.33	8.39	6.65
	NetScience	12.02	6.71	7.73	10.46	9.22	**6**.**10**
	Celegans	6.87	5.96	5.93	7.67	7.86	**5**.**27**
	Euroroads	26.12	10.20	16.90	27.07	32.59	**9**.**97**
0.75	BA model(1)	7.62	4.89	**4**.**58**	9.34	8.04	4.84
	BA model (2)	7.48	6.76	6.24	8.14	7.05	**5**.**66**
	BA model (3)	6.35	6.95	6.28	7.49	7.08	**5**.**64**
	BA model (4)	7.18	6.77	6.54	8.63	6.74	**5**.**84**
	BA model (5)	5.10	6.38	5.86	7.58	5.29	**4**.**98**
	BA model (6)	5.67	6.56	5.98	7.26	6.47	**5**.**20**
	WS model (1)	6.34	**0**.**99**	1.17	11.27	6.79	2.19
	WS model (2)	7.04	1.22	**1**.**05**	10.35	8.44	2.89
	WS model (3)	7.84	**1**.**66**	1.74	10.53	11.56	3.55
	WS model (4)	6.99	1.79	**0**.**83**	10.56	11.92	3.86
	WS model (5)	6.52	1.74	**1**.**49**	10.73	12.29	3.07
	Dolphins	6.98	7.32	5.42	10.10	7.73	**4**.**85**
	Lesmis	7.27	5.78	4.65	10.61	5.81	**4**.**29**
	PDZBase	10.69	5.13	5.39	13.12	9.59	**4**.**82**
	USAirlines	7.31	**4**.**40**	4.91	7.21	7.96	5.53
	NetScience	11.81	5.35	5.82	9.47	7.22	**4**.**98**
	Celegans	6.92	3.72	**3**.**52**	6.76	7.87	4.34
	Euroroads	23.77	7.31	9.32	28.66	14.91	**6**.**67**

The values with bold font are the optimal values

#### Definition 2

Measuring Infection Time. Given an arbitrary $\mathcal {G}=\left( \mathcal {V},\mathcal {E},{\varvec{\theta }}\right)$ and an observers set $\mathcal {O}=\left\{ o_k\right\} ^{\mathcal {K}}_{k=1}$. The Measuring Infection Time of $\mathcal {O}$ in ${\mathcal {T}_{{\text {DIS}},s}}$ is defined as:

3 $$\begin{aligned} {{\textbf {T}}}_{\mathcal {T}_{{\text {DIS}},s}}=\left\{ t_{v_{o_k}}\right\} ^{\mathcal {K}}_{k=1} \end{aligned}$$

where $\mathcal {T}_{{\text {DIS}},s}$ is a relaxed DIS spanning tree of $\mathcal {G}$ with a node *s* as root, $s\in \mathcal {V}$. $t_{v_{o_k}}$ denotes the Measuring Infection Time of node $v_{o_k}$; $v_{o_k}$ is the node with the node number corresponding to the observer $o_k$.

4 $$\begin{aligned} t_{v_{o_k}}= {\left\{ \begin{array}{ll} t^*,&{}v_{o_k}=s\\ t^*+{\sum }_{j\in p\left( s,v_{o_k}\right) }\theta _j,&{}v_{o_k}\ne s \end{array}\right. } \end{aligned}$$

where $t^*$ is the unknown start time. *s* is the root of ${\mathcal {T}_{{\text {DIS}},s}}$. $p\left( s,v_{o_k}\right)$ denotes the path from *s* to $v_{o_k}$ in the ${\mathcal {T}_{{\text {DIS}},s}}$. $\theta _j$ denotes the propagation delay.

**Fig. 9:**
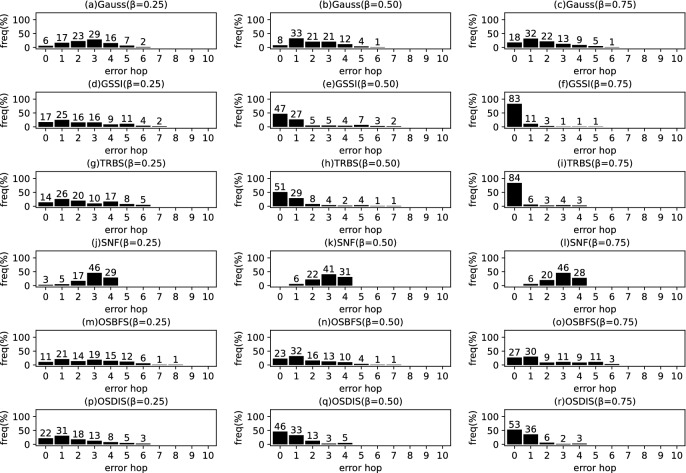
The results of Gauss, GSSI, TRBS, SNF, OSBFS and OSDIS algorithms applied on WS model (1). Each sub-figure is obtained by 100 runs

In fact, in the current fixed ${\mathcal {T}_{{\text {DIS}},s}}$ with *s* as root, the *s* is assumed to be the propagation source. Thus, $t^*$ minimizes the difference between ${{\textbf {T}}}_{\mathcal {O}}$ and ${{\textbf {T}}}_{\mathcal {T}_{{\text {DIS}},s}}$; $t^*$ can be estimated by the following function.

5 $$\begin{aligned} \begin{aligned} \hat{t^*}=&\arg \min \sum _{k=1}^\mathcal {K}\left( t_{v_{o_k}}-t_{o_k}\right) ^2\\ =&\arg \min \sum _{k=1}^\mathcal {K}\left( t^*+\sum _{j\in p\left( s,v_{o_k}\right) }\theta _j-t_{o_k}\right) ^2 \end{aligned} \end{aligned}$$

where $t^*\in \left[ 0,z\right]$, $z\le \sum \theta$, $\theta \in {\varvec{\theta }}$. $t_{v_{o_k}}$ denotes the Measuring Infection Time of $v_{o_k}$. $t_{o_k}$ denotes the Infection Timing information recorded of $o_k$.

**Fig. 10:**
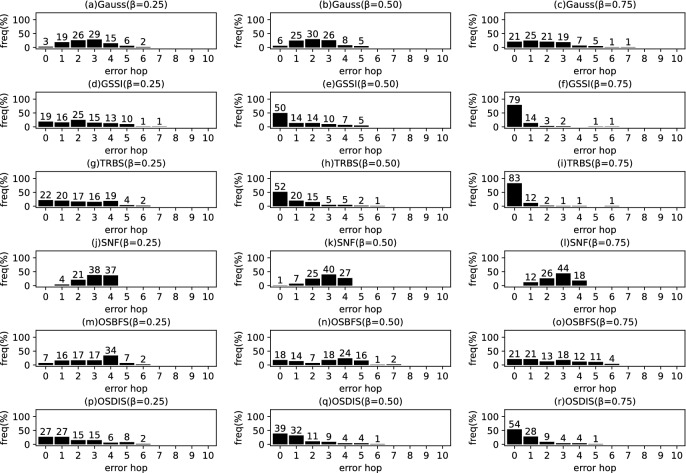
The results of Gauss, GSSI, TRBS, SNF, OSBFS and OSDIS algorithms applied on WS model (2). Each sub-figure is obtained by 100 runs

**Fig. 11:**
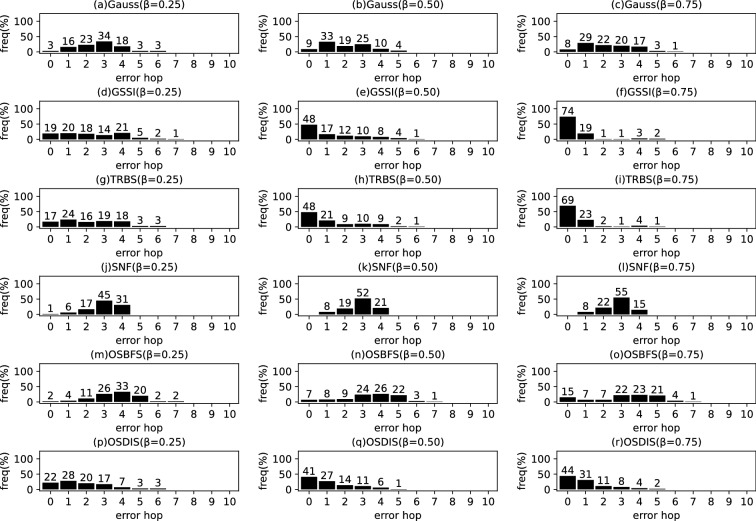
The results of Gauss, GSSI, TRBS, SNF, OSBFS and OSDIS algorithms applied on WS model (3). Each sub-figure is obtained by 100 runs

#### Definition 3

Infection Time Similarity is defined as:

6 $$\begin{aligned} \begin{aligned} \varvec{\mathcal {S}}\left( {{\textbf {T}}}_{\mathcal {O}},{{\textbf {T}}}_{\mathcal {T}_{{\text {DIS}},s}}\right) =&\frac{1}{1+\mathcal {D}\left( {{\textbf {T}}}_{\mathcal {O}},{{\textbf {T}}}_{\mathcal {T}_{{\text {DIS}},s}}\right) }\\ =&\frac{1}{1+\left( \sum \nolimits _{k=1}^\mathcal {K}\vert t_{v_{o_k}}-t_{o_k}\vert ^{2}\right) ^{1/2}} \end{aligned} \end{aligned}$$

where $\mathcal {D}\left( {{\textbf {T}}}_{\mathcal {O}},{{\textbf {T}}}_{\mathcal {T}_{{\text {DIS}},s}}\right)$ denotes the Euclidean distance (Rui and Wunsch 2005) between the ${{\textbf {T}}}_{\mathcal {O}}$ (Definition 1) and the ${{\textbf {T}}}_{\mathcal {T}_{{\text {DIS}},s}}$ (Definition 2). $\varvec{\mathcal {S}}\left( {{\textbf {T}}}_{\mathcal {O}},{{\textbf {T}}}_{\mathcal {T}_{{\text {DIS}},s}}\right) \in \left( 0,1\right]$.

#### Definition 4

Observation Infection Time Order. Given an arbitrary $\mathcal {G}=\left( \mathcal {V},\mathcal {E},{\varvec{\theta }}\right)$ and an observers set $\mathcal {O}=\left\{ o_k\right\} ^{\mathcal {K}}_{k=1}$. The Observation Time Order of $\mathcal {O}$ is defined as an ordered observers sequence ${{\textbf {TO}}}_{\mathcal {O}}=\left\langle o_i\right\rangle ^\mathcal {K}_{i=1}$, in which each $o_i$ is sorted by ascending order according to the Infection Timing information (denoted by $t_{o_i}$) recorded in $o_i$. For any pair of observers $o_i,o_{i+1}\in {{\textbf {TO}}}_{\mathcal {O}}$, there is $t_{o_i}\le t_{o_{i+1}}$.

#### Definition 5

Measuring Infection Time Order. Given an arbitrary $\mathcal {G}=\left( \mathcal {V},\mathcal {E},{\varvec{\theta }}\right)$ and an observers set $\mathcal {O}=\left\{ o_k\right\} ^{\mathcal {K}}_{k=1}$. The Measuring Infection Time Order of $\mathcal {O}$ on $\mathcal {T}_{{\text {DIS}},s}$ is defined as an ordered nodes sequence ${{\textbf {TO}}}_{\mathcal {T}_{{\text {DIS}},s}}=\left\langle v_{o_k}\right\rangle ^\mathcal {K}_{k=1}$, in which each $v_{o_k}\in {{\textbf {TO}}}_{\mathcal {T}_{{\text {DIS}},s}}$ is sorted by ascending order according to the Measuring Infection Time $t_{v_{o_k}}$ (Definition 2). For any pair of nodes $v_{o_k},v_{o_{k+1}}\in {{\textbf {TO}}}_{\mathcal {T}_{{\text {DIS}},s}}$, there is $t_{v_{o_k}}\le t_{v_{o_{k+1}}}$.

#### Definition 6

Infection Time Order Similarity is defined as:

7 $$\begin{aligned} \varvec{\mathcal {S}}\left( {{\textbf {TO}}}_{\mathcal {O}},{{\textbf {TO}}}_{\mathcal {T}_{{\text {DIS}},s}}\right) =\frac{1+\tau \left( {{\textbf {TO}}}_{\mathcal {O}},{{\textbf {TO}}}_{\mathcal {T}_{{\text {DIS}},s}}\right) }{2} \end{aligned}$$

where $\tau$ denotes the correlation coefficient defined in reference (Kendall 1938); the details can be found in Appendix A. $\tau$ is mainly used to measure the concordance between the ${{\textbf {TO}}}_{\mathcal {O}}$ (Definition 4) and the ${{\textbf {TO}}}_{\mathcal {T}_{{\text {DIS}},s}}$ (Definition 5). $\varvec{\mathcal {S}}\left( {{\textbf {TO}}}_{\mathcal {O}},{{\textbf {TO}}}_{\mathcal {T}_{{\text {DIS}},s}}\right) \in \left[ 0,1\right]$.

The properties related to the Infection Time Similarity (Definition 3) and the Infection Time Order Similarity (Definition 6) can be found in Appendix B.

*Example:* In Fig. 1, Fig. 1b shows a relaxed DIS spanning tree of the network shown in Fig. 1a. $\mathcal {O}=\left\{ o_1, o_2, o_3\right\}$, $o_1$, $o_2$ and $o_3$ correspond to nodes 2, 3 and 6, respectively. According to Definition 1, for ${{\textbf {T}}}_{\mathcal {O}}$, $t_2=4$, $t_3=7$, $t_6=6$. When the current root is node 1, according to Eq. 5, $t^*=1$. According to Definition 2, for ${{\textbf {T}}}_{\mathcal {T}_{{\text {DIS}},s}}$, $t_2=4$, $t_3=7$, $t_6=6$. Then, according to Definition 3, we have $\varvec{\mathcal {S}}\left( {{\textbf {T}}}_{\mathcal {O}},{{\textbf {T}}}_{\mathcal {T}_{{\text {DIS}},s}}\right) =1$. According to Definition 4, ${{\textbf {TO}}}_{\mathcal {O}}=\left\langle 2,6,3\right\rangle$. According to Definition 5, ${{\textbf {TO}}}_{\mathcal {T}_{{\text {DIS}},s}}=\left\langle 2,6,3\right\rangle$. Further, with Definition 6, we have $\varvec{\mathcal {S}}\left( {{\textbf {TO}}}_{\mathcal {O}},{{\textbf {TO}}}_{\mathcal {T}_{{\text {DIS}},s}}\right) =1$.

**Fig. 12:**
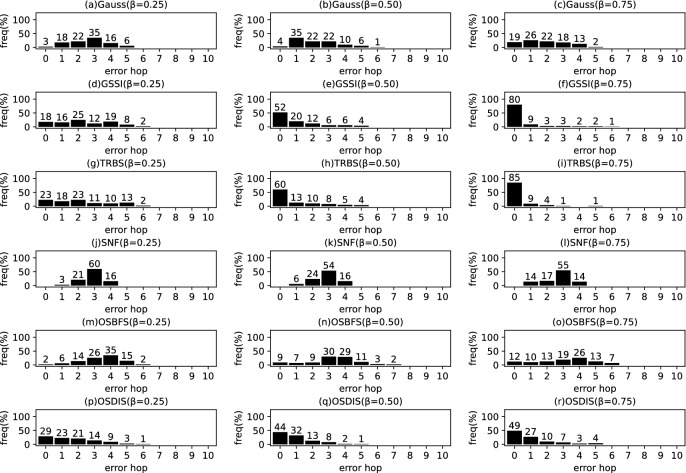
The results of Gauss, GSSI, TRBS, SNF, OSBFS and OSDIS algorithms applied on WS model (4). Each sub-figure is obtained by 100 runs

**Fig. 13:**
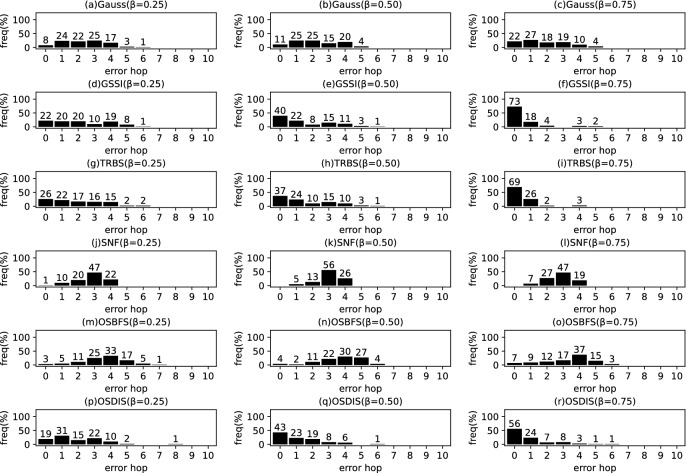
The results of Gauss, GSSI, TRBS, SNF, OSBFS and OSDIS algorithms applied on WS model (5). Each sub-figure is obtained by 100 runs

**Fig. 14:**
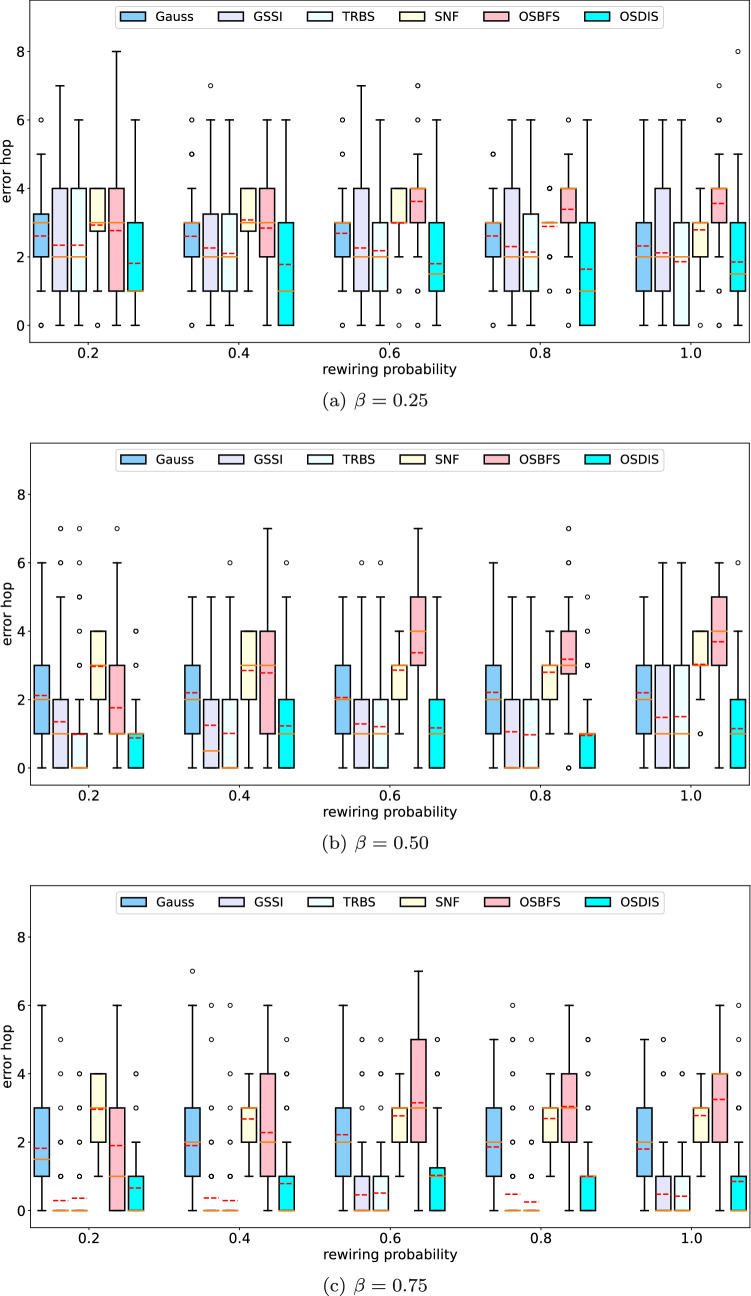
The IQR (box-plot) of error hop of Gauss, GSSI, TRBS, SNF, OSBFS and OSDIS algorithms on the WS models generated by different rewiring probabilities. The orange line and red line in each box denote the median and average error hop (also shown in Table 6), respectively (color figure online)

### Locating the propagation source

By combining the Infection Time Similarity (Definition 3) and the Infection Time Order Similarity (Definition 6), the source estimator in Eq. 2 can be written as follows:

8 $$\begin{aligned} \begin{aligned} \hat{s}&=\mathop {\arg \max }_{s\in \mathcal {V}}\varvec{\mathcal {S}}\left( \mathcal {O}_{\mathcal {T}_{s^*}}, \mathcal {O}_{\mathcal {T}_{{\text {DIS}},s}}\right) \\&=\mathop {\arg \max }_{s\in \mathcal {V}}\left( \varvec{\mathcal {S}}\left( {{\textbf {T}}}_{\mathcal {O}}, {{\textbf {T}}}_{\mathcal {T}_{{\text {DIS}},s}}\right) \times \varvec{\mathcal {S}}\left( {{\textbf {TO}}}_{\mathcal {O}}, {{\textbf {TO}}}_{\mathcal {T}_{{\text {DIS}},s}}\right) \right) \end{aligned} \end{aligned}$$

where $\varvec{\mathcal {S}}\left( {{\textbf {T}}}_{\mathcal {O}},{{\textbf {T}}}_{\mathcal {T}_{{\text {DIS}},s}}\right)$ and $\varvec{\mathcal {S}}\left( {{\textbf {TO}}}_{\mathcal {O}},{{\textbf {TO}}}_{\mathcal {T}_{{\text {DIS}},s}}\right)$ are defined in Definition 3 and Definition 6, respectively.

Based on Eq. 8, we propose a novel source locating method, termed as OSDIS algorithm, which is summarized in Algorithm 2.

Algorithm 2 analysis: The $E\left( \mathcal {T}\right)$ declared in line 2 is an edge set. Lines 3–19 are used for traversing the $\mathcal {G}$ by the relaxed DIS with current node *s* as root and recording the eligible edges into $E\left( \mathcal {T}\right)$. Lines 3–19 require $O\left( \vert \mathcal {V}\vert ^2\right)$ computations in the worst case. Line 20 requires $O\left( \vert \mathcal {V}\vert +\vert \mathcal {E}\vert \right)$ computations, which can be reduced to $O\left( \vert \mathcal {E}\vert \right)$. In lines 21–22, the $\mathcal {T}$ obtained in line 20 will be marked as a relaxed DIS spanning tree $\mathcal {T}_{{\text {DIS}},s}$ if and only if $\vert E\left( \mathcal {T}\right) \vert ==\vert \mathcal {V}\vert -1$. Line 23 requires $O\left( \mathcal {K}\right)$ computations. Line 24 requires $O\left( \vert \mathcal {V}\vert ^2+z\vert \mathcal {V}\vert \mathcal {K}\right)$ computations (*z* can be found in Eq. 5). Lines 25–26 require $O\left( \vert \mathcal {V}\vert \right)$ and $O\left( \mathcal {K}^2\right)$ computations, respectively. Both lines 27 and 28 require $O\left( \mathcal {K}\log \mathcal {K}\right)$ computations. Line 29 requires $O\left( \mathcal {K}^2\right)$ computations. Finally, each node $s\in \mathcal {V}$ will be used as root to construct different $\mathcal {T}_{{\text {DIS}},s}$. Thus, the time complexity of Algorithm 2 is $O\left( \vert \mathcal {V}\vert ^3+z\vert \mathcal {V}\vert ^2\mathcal {K}\right)$.
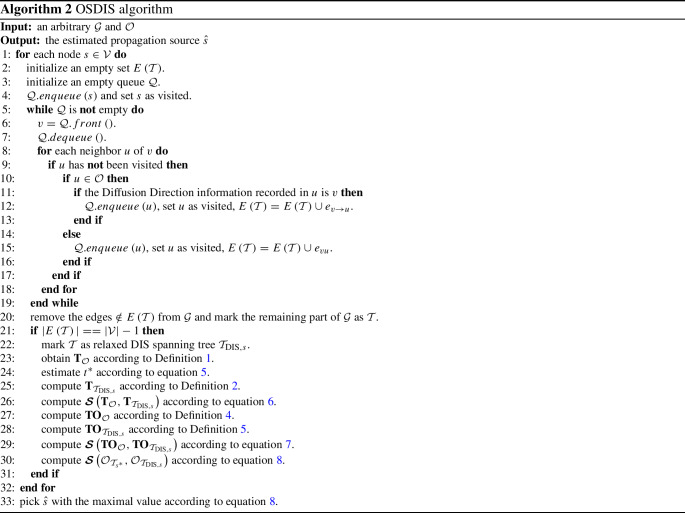


**Fig. 15:**
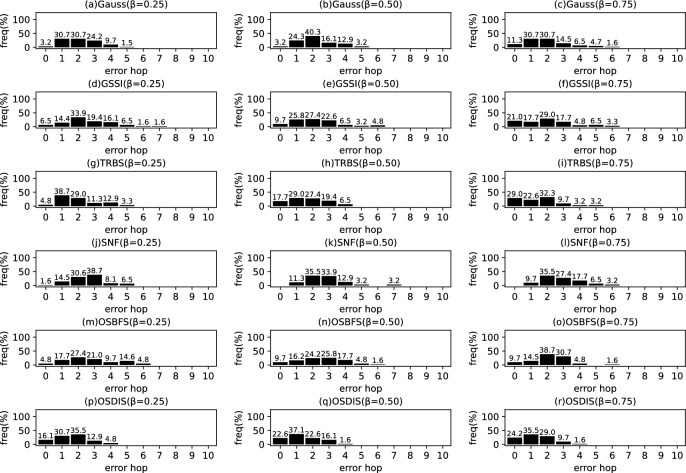
The results of Gauss, GSSI, TRBS, SNF, OSBFS and OSDIS algorithms applied on Dolphins network. Each sub-figure is obtained by 62 runs

**Fig. 16:**
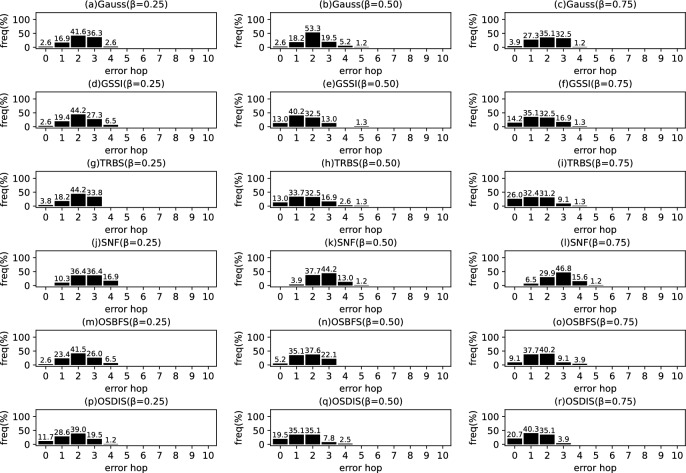
The results of Gauss, GSSI, TRBS, SNF, OSBFS and OSDIS algorithms applied on Lesmis network. Each sub-figure is obtained by 77 runs

**Fig. 17:**
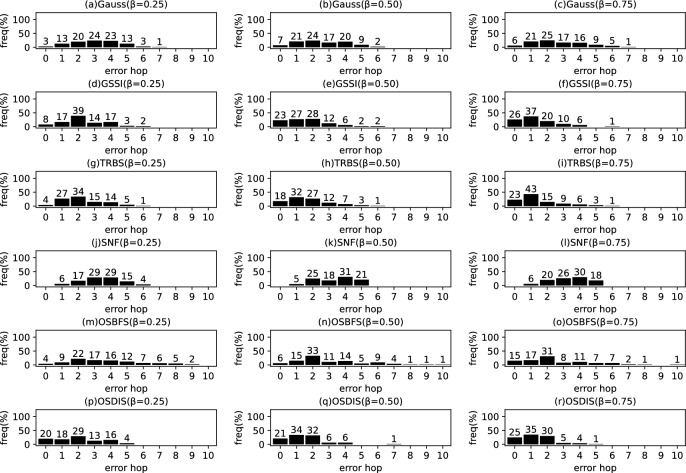
The results of Gauss, GSSI, TRBS, SNF, OSBFS and OSDIS algorithms applied on PDZBase network. Each sub-figure is obtained by 100 runs

**Fig. 18:**
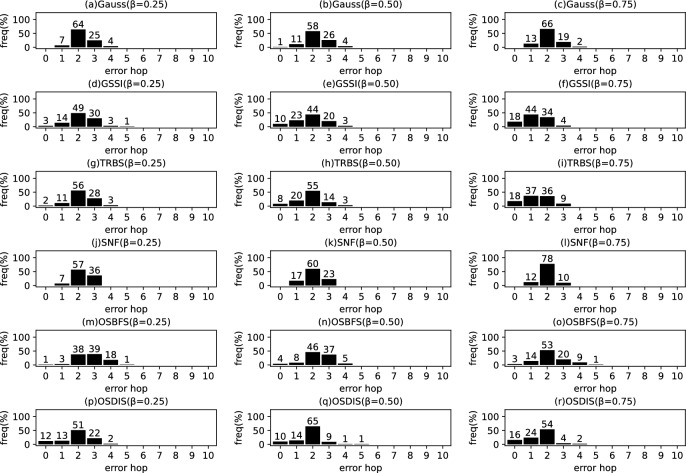
The results of Gauss, GSSI, TRBS, SNF, OSBFS and OSDIS algorithms applied on USAirlines network. Each sub-figure is obtained by 100 runs

**Fig. 19:**
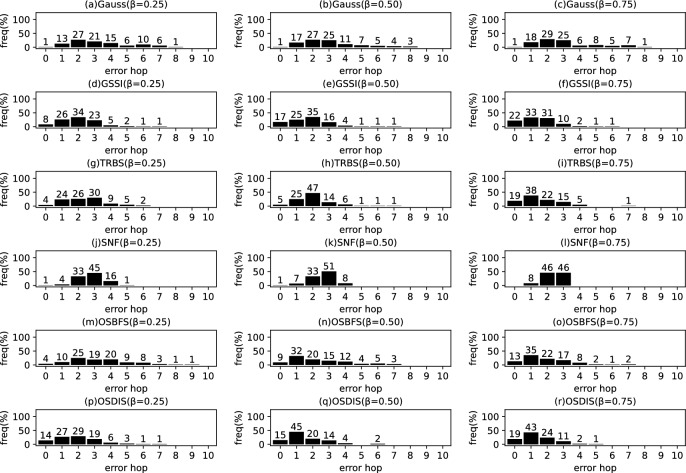
The results of Gauss, GSSI, TRBS, SNF, OSBFS and OSDIS algorithms applied on NetScience network. Each sub-figure is obtained by 100 runs

**Fig. 20:**
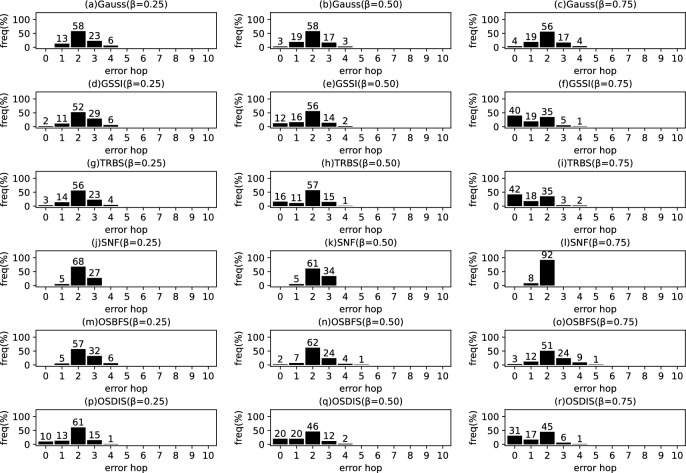
The results of Gauss, GSSI, TRBS, SNF, OSBFS and OSDIS algorithms applied on Celegans network. Each sub-figure is obtained by 100 runs

**Fig. 21:**
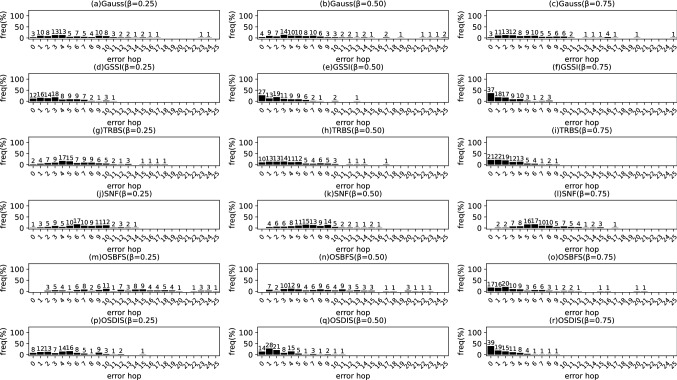
The results of Gauss, GSSI, TRBS, SNF, OSBFS and OSDIS algorithms applied on Euroroads network. Each sub-figure is obtained by 100 runs

## Experimental evaluation

To validate the feasibility and effectiveness of the OSDIS algorithm, it is compared with other four state-of-the-art methods on a series of synthetic and real networks. The four methods include the Gauss algorithm (Pinto et al. 2012), GSSI algorithm (Tang et al. 2018), TRBS algorithm (Shen et al. 2016) and SNF algorithm (Wang and Sun 2020). Besides, since the OSDIS algorithm is based on the relaxed DIS heuristic, to show its advantage, we also define an algorithm, denoted by OSBFS, in which the relaxed DIS heuristic is replaced by the breadth-first search (BFS) heuristic. Totally, six algorithms are compared in the experiments. Their time complexity is shown in Table 2. Similar to the reference (Yang et al. 2020), the performance of a source locating algorithm is mainly evaluated by the precision (the precise locating ratio, i.e., the proportion of 0 error hop), the average error hop and the average error delay. For the precision, the higher the value is, the better the algorithm is. For the average error hop and the average error delay, the smaller the value is, the better the algorithm is.

*Running environment* Hardware: Dell R740 with 2 Intel$^{\small {\textcircled {\tiny {R}}}}$Xeon$^{\small {\textcircled {\tiny {R}}}}$ gold 6254 CPU, 1T RAM. Software: Cygwin 3.0.7 + Eclipse Cpp2019 + igraph C 0.7.1 + Eigen/Dense (used for running algorithms). R 64 $\times$ 3.3.3 + igraph R 1.2.1 (used for generating synthetic networks).

*Datasets* The six algorithms are evaluated on a series of synthetic and real networks. The synthetic networks include the scale-free (BA) model (Barabasi and Albert 1999) and the small-world (WS) model (Watts and Strogatz 1998). Totally, six BA models with different powers of the preferential attachment and five WS models with different rewiring probabilities are generated, respectively. The detailed parameters for generating these synthetic networks are shown in Table 3. The real networks are selected from different fields, which can be obtained from the Koblenz Network Collection (Kunegis 2013) and the Network Data Repository (Rossi and Ahmed 2015) for free. All the real networks are shown in Table 4. The topology properties of the used networks are shown in Table 5.

*Parameters setting* Given an arbitrary graph $\mathcal {G}$, the propagation delays set ${\varvec{\theta }}$ are independent identically distributed (i.i.d) random variables with Gaussian distribution $\mathcal {N}\left( \mu , \sigma ^2 \right)$, $\mu$ and $\sigma ^2$ are known (Pinto et al. 2012; Paluch et al. 2018). We set $\mu /\sigma =4$. The diffusion model follows the one introduced in Sect. 3. To investigate the impact of different propagation ratios (denoted by $\beta$) on the performance of the source locating algorithms, we set $\beta =0.25$, $\beta =0.50$ and $\beta =0.75$, respectively. Additionally, to compare with the GSSI algorithm (Tang et al. 2018), we set $s^*\notin \mathcal {O}$. Generally, in reality, to save the cost, the number of the observers will be far less than the size of $\mathcal {G}$. Thus, we randomly select $5\%$ nodes as the observers in each network.

**Table 8:** The average running time ratios between other five algorithms and the OSDIS on different networks

Network	$\frac{t(\textrm{Gauss})}{t(\textrm{OSDIS})}$	$\frac{t(\textrm{GSSI})}{t(\textrm{OSDIS})}$	$\frac{t(\textrm{TRBS})}{t(\textrm{OSDIS})}$	$\frac{t(\textrm{SNF})}{t(\textrm{OSDIS})}$	$\frac{t(\textrm{OSBFS})}{t(\textrm{OSDIS})}$
BA model (1)	59.52	10.27	0.0004	0.03	0.68
BA model (2)	52.24	9.61	0.0003	0.02	0.58
BA model (3)	55.94	9.77	0.0004	0.02	0.39
BA model (4)	54.16	9.62	0.0004	0.02	0.31
BA model (5)	56.71	9.84	0.0006	0.02	0.24
BA model (6)	55.22	9.83	0.0006	0.02	0.24
WS model (1)	43.98	7.56	0.0007	0.03	1.04
WS model (2)	54.13	9.29	0.0004	0.03	1.00
WS model (3)	57.22	10.00	0.0005	0.04	0.86
WS model (4)	59.48	10.34	0.0003	0.04	0.83
WS model (5)	61.03	10.50	0.0003	0.06	0.80
Dolphins	42.52	16.81	0.0067	0.29	0.90
Lesmis	30.69	9.52	0.0100	0.11	0.83
PDZBase	60.84	11.67	0.0007	0.05	0.60
USAirlines	75.98	11.71	0.0002	0.02	0.68
NetScience	104.62	15.35	0.0001	0.02	1.18
Celegans	138.81	19.98	0.0001	0.01	0.69
Euroroads	1342.74	180.82	$\approx 0$	0.02	1.06

$t\left( \cdot \right)$: the running time of an algorithm

### Experimental results on the synthetic networks

Figures 2, 3, 4, 5, 6, 7 show the precision (the precise locating ratio, i.e., the proportion of 0 error hop) of the six algorithms on a series of BA models. From Figs. 2, 3, 4, 5, 6, 7, we can see that, when $\beta =0.25$, $\beta =0.5$ and $\beta =0.75$, the OSDIS algorithm generally exposes the best precision on all the six BA models, i.e., the OSDIS has a higher proportion in 0 error hop than other five algorithms. Only when $\beta =0.75$, the OSDIS is inferior to the GSSI and TRBS on BA model (1), but outperforms other three algorithms. From Table 6, we know that, when $\beta =0.25$, $\beta =0.5$ and $\beta =0.75$, the OSDIS is better than other five algorithms in the average error hop on all the six BA models. Only when $\beta =0.5$, the OSDIS is a litter inferior to OSBFS on BA model (5), but superior to other four algorithms. Meanwhile, in Fig. 8, we plot interquartile range (IQR) to show the distribution regions of error hop of the six algorithms on the BA models. Additionally, from Table 7, we can see that, when $\beta =0.25$, $\beta =0.5$ and $\beta =0.75$, the OSDIS exposes a better average error delay on all the six BA models. Only when $\beta =0.75$, the OSDIS is inferior to TRBS on BA model (1), but outperforms other four algorithms. In summary, on the BA models, the OSDIS is generally better than other five algorithms in the precision, the average error hop and average error delay.

Figures 9, 10, 11, 12, 13 show the precision (the precise locating ratio, i.e., the proportion of 0 error hop) of the six algorithms on a series of WS models. From Figs. 9, 10, 11, 12, 13, we can see that, when $\beta =0.25$, the OSDIS is superior to other five algorithms in the precision on WS models (1)–(4) (i.e., the OSDIS has a higher proportion in 0 error hop), but only inferior to TRBS and GSSI on WS model (5). When $\beta =0.5$ and $\beta =0.75$, the OSDIS is generally inferior to TRBS and GSSI in the precision on WS models (1)–(5), but superior to other three algorithms. Only when $\beta =0.5$, the OSDIS exposes the best precision on WS model (5). From Tables 6 and 7, we know that, when $\beta =0.25$ and $\beta =0.5$, the OSDIS is generally better than other five algorithms in the average error hop and average error delay on all the five WS models. Only when $\beta =0.5$, the OSDIS is inferior to TRBS on WS model (2). Meanwhile, from Tables 6 and 7, we know that, when $\beta =0.75$, the OSDIS is always inferior to GSSI and TRBS in the average error hop and average error delay, but outperforms other three algorithms. In Fig. 14, we further plot interquartile range (IQR) to show the distribution regions of error hop of the six algorithms on the WS models. In summary, on the WS models, the OSDIS is generally superior to other five algorithms in the precision when $\beta =0.25$ and generally exposes a better performance in the average error hop and average error delay when $\beta =0.25$ and $\beta =0.5$. Thus, the OSDIS is better than other five algorithms in most cases. Obviously, the performance of the OSDIS on the BA models is better than on the WS models.

### Experimental results on the real networks

**Fig. 22:**
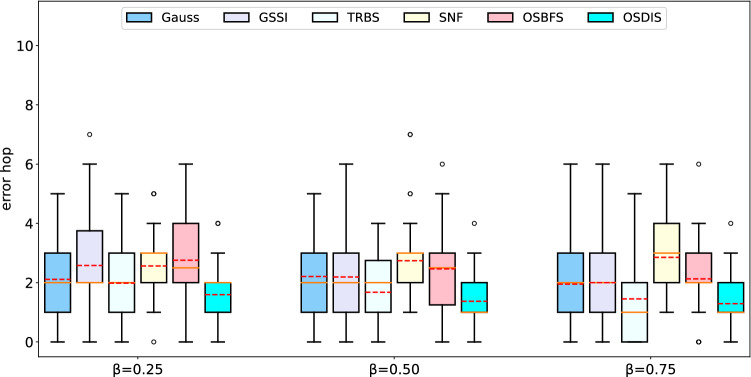
The IQR (box-plot) of error hop of Gauss, GSSI, TRBS, SNF, OSBFS and OSDIS algorithms on Dolphins network. The orange line and red line in each box denote the median and average error hop, respectively (color figure online)

**Fig. 23:**
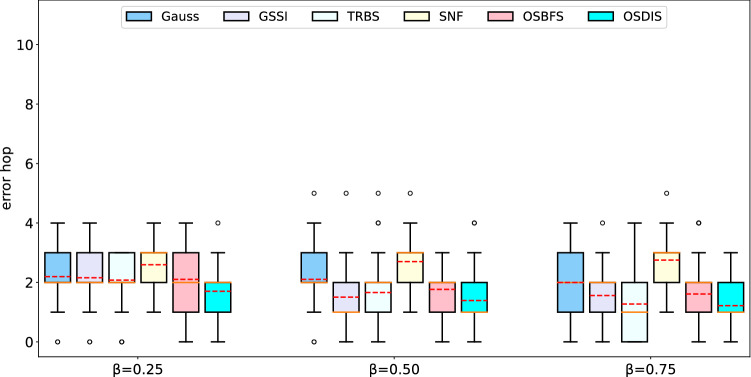
The IQR (box-plot) of error hop of Gauss, GSSI, TRBS, SNF, OSBFS and OSDIS algorithms on Lesmis network. The orange line and red line in each box denote the median and average error hop, respectively (color figure online)

**Fig. 24:**
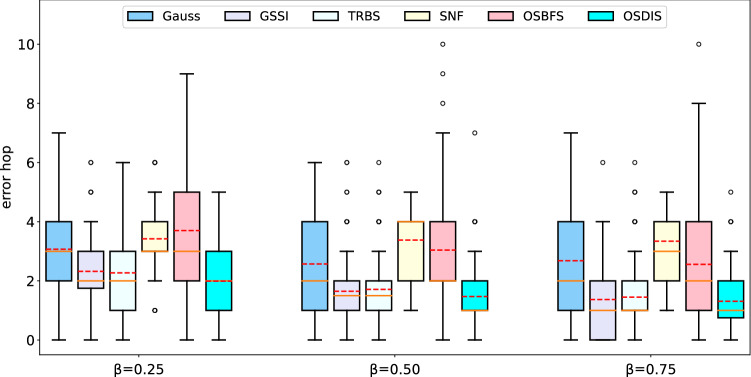
The IQR (box-plot) of error hop of Gauss, GSSI, TRBS, SNF, OSBFS and OSDIS algorithms on PDZBase network. The orange line and red line in each box denote the median and average error hop, respectively (color figure online)

**Fig. 25:**
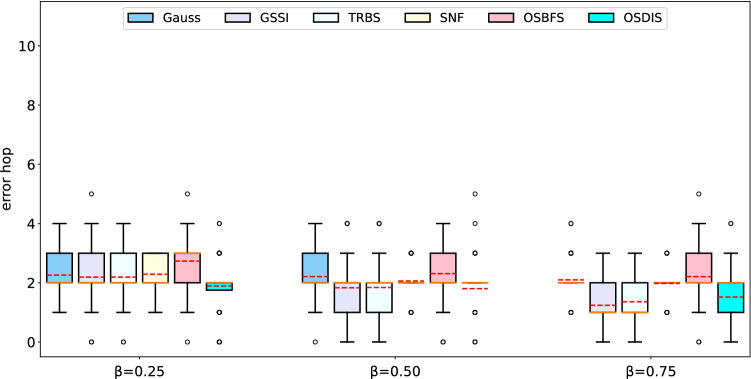
The IQR (box-plot) of error hop of Gauss, GSSI, TRBS, SNF, OSBFS and OSDIS algorithms on USAirlines network. The orange line and red line in each box denote the median and average error hop, respectively (color figure online)

**Fig. 26:**
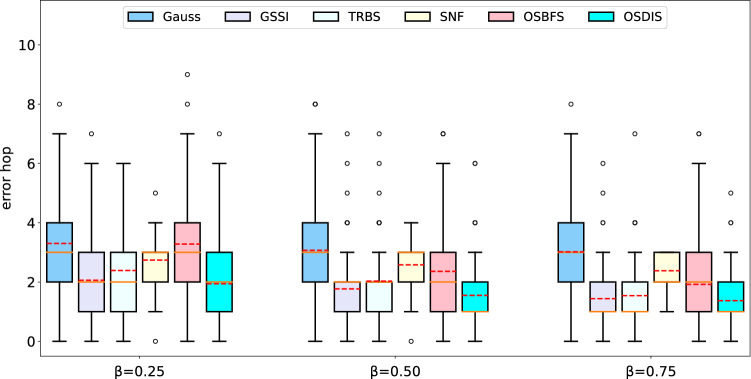
The IQR (box-plot) of error hop of Gauss, GSSI, TRBS, SNF, OSBFS and OSDIS algorithms on NetScience network. The orange line and red line in each box denote the median and average error hop, respectively (color figure online)

**Fig. 27:**
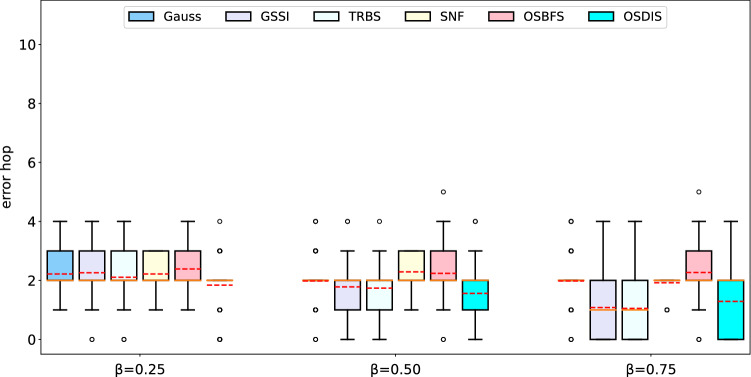
The IQR (box-plot) of error hop of Gauss, GSSI, TRBS, SNF, OSBFS and OSDIS algorithms on Celegans network. The orange line and red line in each box denote the median and average error hop, respectively (color figure online)

**Fig. 28:**
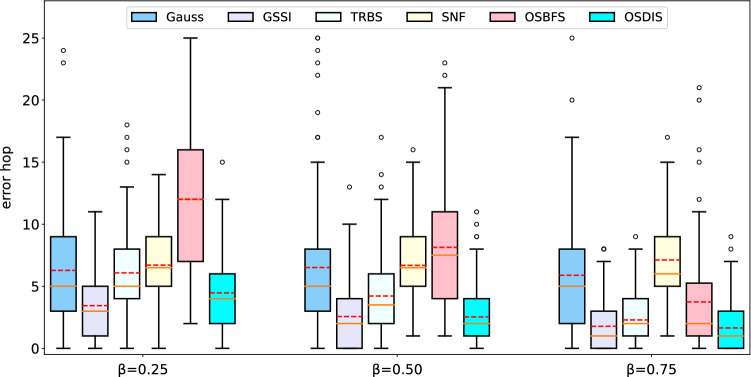
The IQR (box-plot) of error hop of Gauss, GSSI, TRBS, SNF, OSBFS and OSDIS algorithms on Euroroads network. The orange line and red line in each box denote the median and average error hop, respectively (color figure online)

In this subsection, we further validate the performance of the six algorithms on the real networks. Figures 15, 16, 17, 18, 19, 20, 21 show the precision (the precise locating ratio, i.e., the proportion of 0 error hop) of the six algorithms on the real networks. When $\beta =0.25$, from Figs. 15, 16, 17, 18, 19, 20, 21, we can see that the OSDIS generally exposes the best precision (i.e., the OSDIS has a higher proportion in 0 error hop) on all the real networks, except for Euroroads network on which the OSDIS is only inferior to GSSI, but superior to other four algorithms. By combining with Tables 6 and 7, we know that the OSDIS is also superior to other five algorithms in the average error hop and the average error delay on all the real networks, except for Euroroads network on which the OSDIS is only inferior to GSSI, but superior to other four algorithms. When $\beta =0.5$, from Figs. 15, 16, 17, 18, 19, 20, 21, we know that the OSDIS exposes the best precision (i.e., the OSDIS has a higher proportion in 0 error hop) on Dolphins, Lesmis, USAirlines and Celegans networks. Meanwhile, the OSDIS is only inferior to GSSI in the precision on PDZBase, NetScience and Euroroads networks, but superior to other four algorithms. By combining with Tables 6 and 7, we can see that the OSDIS generally outperforms other five algorithms in the average error hop and the average error delay on the real networks. Only on USAirlines network, the OSDIS is inferior to GSSI and TRBS in the average error delay. When $\beta =0.75$, from Figs. 15, 16, 17, 18, 19, 20, 21, we know that, except for Euroroads network, the OSDIS is generally inferior to TRBS or GSSI in the precision on the real networks. By combining with Tables 6 and 7, we can see that the OSDIS outperforms other five algorithms in the average error hop and average error delay on Dolphins, Lesmis, PDZBase, NetScience and Euroroads networks, but is inferior to GSSI and TRBS on USAirlines and Celegans networks. Meanwhile, in Appendix C Figs. 22, 23, 24, 25, 26, 27, 28, we plot interquartile range (IQR) to further show the distribution regions of error hop of the six algorithms on the real networks. In summary, on the real networks, the OSDIS is generally superior to other five algorithms in the precision, the average error hop and the average error delay when $\beta =0.25$ and $\beta =0.5$, but inferior to GSSI and TRBS when $\beta =0.75$. Thus, the OSDIS is better than other five algorithms in most cases.

Overall, in the precision, the average error hop and average error delay, the OSDIS outperforms other five algorithms on the BA models and in most cases is superior to other five algorithms on the WS models and real networks. In a few cases, the OSDIS is only inferior to GSSI and TRBS, but superior to other three algorithms. Thus, the OSDIS is a feasible and effective method in accurately locating the propagation source. Meanwhile, on the BA models, WS models and real networks, the OSDIS obviously outperforms the OSBFS, which indicates that the relaxed DIS heuristic outperforms the BFS heuristic in locating the propagation source.

The average error hops of the six algorithms on different networks are shown in Table 6. The average error delay is shown in Table 7. The average running time ratios between other five algorithms and the OSDIS on all networks are shown in Table 8. From Table 8, we can see that the efficiency of the OSDIS is inferior to the TRBS and SNF, similar with the OSBFS, and superior to the Gauss and GSSI.

## Conclusion

In this paper, we locate the propagation source by utilizing both of the diffusion direction information and the infection time information of the observers. We introduce a relaxed direction-induced search (DIS) to utilize the diffusion direction information of the observers to approximate the actual diffusion tree on a network. Based on the relaxed DIS, we further utilize the infection time information of the observers to define two kinds of observer-based similarity measures, including the Infection Time Similarity and the Infection Time Order Similarity. With the two kinds of similarity measures and the relaxed DIS, a source locating method termed as OSDIS is proposed. The feasibility and effectiveness of the OSDIS are validated on a series of synthetic and real networks. Meanwhile, the experimental results also show that the relaxed DIS heuristic outperforms the BFS heuristic in propagation source locating. The current OSDIS is only developed for single source locating. In the future work, we will study the OSDIS-based multi-sources locating method.

## Data Availability

Enquiries about data availability should be directed to the authors.

## A. Correlation coefficient

The correlation coefficient $\tau$ defined in reference (Kendall 1938) considers a set of joint ranks from two sequence ${{\textbf {X}}}$ and ${{\textbf {Y}}}$. For arbitrary $x_i, x_j\in {{\textbf {X}}}$, $y_i,y_j\in {{\textbf {Y}}}$, any pair of two-tuples $\left( x_i,y_i\right)$ and $\left( x_j,y_j\right)$ $\left( i\ne j\right)$ are said to be concordant if both $x_i>x_j$ and $y_i>y_j$ or if both $x_i<x_j$ and $y_i<y_j$. They are said to be discordant if both $x_i>x_j$ and $y_i<y_j$ or if both $x_i<x_j$ and $y_i>y_j$. If $x_i=x_j$ or $y_i=y_j$, the pair is neither concordant nor discordant. $\tau$ is defined as:

9 $$\begin{aligned} \tau =\frac{2\times \left( n_c-n_d\right) }{n(n-1)} \end{aligned}$$

where $n_c$ and $n_d$ denote the number of concordant and discordant pairs, respectively. $\tau \in \left[ -1,1\right]$.

## B. Properties

### Property 1

Suppose that the propagation ratio of the SI model is $\beta =1$, for $\mathcal {G}=\left( \mathcal {V},\mathcal {E},{\varvec{\theta }}\right)$ and $\mathcal {O}=\left\{ o_k\right\} ^{\mathcal {K}}_{k=1}$, suppose $\mathcal {O}=\mathcal {V}$, then

10 $$\begin{aligned} \varvec{\mathcal {S}}\left( {{\textbf {T}}}_{\mathcal {O}},{{\textbf {T}}}_{\mathcal {T}_{{\text {DIS}},s}}\right) =1 \end{aligned}$$

### Proof

By Eq. 6, the proof of Eq. 10 can be converted into the proof about $\mathcal {D}\left( {{\textbf {T}}}_{\mathcal {O}},{{\textbf {T}}}_{\mathcal {T}_{{\text {DIS}},s}}\right) =0$. Firstly, since $\beta =1$, on the original $\mathcal {G}$, each node $o_k$ is first time infected along the weighted shortest path between $s^*$ and $o_k$. Meanwhile, the Infection Timing information can be recorded in $o_k$, denoted by $t_{o_k}$ (Definition 1). Secondly, since $\mathcal {O}=\mathcal {V}$, by combining with Algorithm 1, we know that the $\mathcal {T}_{{\text {DIS}},s}$ is the actual diffusion tree corresponding to the first time each node in $\mathcal {G}$ gets infected. Thus, for the Measuring Infection Time of each $v_{o_k}$ (denoted by $t_{v_{o_k}}$, Definition 2), there is $t_{v_{o_k}}=t_{o_k}$. According to the $\mathcal {D}\left( {{\textbf {T}}}_{\mathcal {O}},{{\textbf {T}}}_{\mathcal {T}_{{\text {DIS}},s}}\right)$ in Definition 3, there is $\mathcal {D}\left( {{\textbf {T}}}_{\mathcal {O}},{{\textbf {T}}}_{\mathcal {T}_{{\text {DIS}},s}}\right) =0$. Property 1 is proved. $\square$

### Property 2

Suppose that the propagation ratio of the SI model is $\beta =1$, for $\mathcal {G}=\left( \mathcal {V},\mathcal {E},{\varvec{\theta }}\right)$ and $\mathcal {O}=\left\{ o_k\right\} ^{\mathcal {K}}_{k=1}$, suppose $\mathcal {O}=\mathcal {V}$, then

11 $$\begin{aligned} \varvec{\mathcal {S}}\left( {{\textbf {TO}}}_{\mathcal {O}},{{\textbf {TO}}}_{\mathcal {T}_{{\text {DIS}},s}}\right) =1 \end{aligned}$$

### Proof

By Eq. 7, the proof of Eq. 11 can be converted into the proof about $\tau \left( {{\textbf {TO}}}_{\mathcal {O}},{{\textbf {TO}}}_{\mathcal {T}_{{\text {DIS}},s}}\right) =1$. From Property 1, we know that, since $\mathcal {O}=\mathcal {V}$, then we get $\varvec{\mathcal {S}}\left( {{\textbf {T}}}_{\mathcal {O}},{{\textbf {T}}}_{\mathcal {T}_{{\text {DIS}},s}}\right) =1$. For ${{\textbf {T}}}_{\mathcal {O}}=\left\{ t_{o_k}\right\} ^{\mathcal {K}}_{k=1}$ and ${{\textbf {T}}}_{\mathcal {T}_{{\text {DIS}},s}}=\left\{ t_{v_{o_k}}\right\} ^\mathcal {K}_{k=1}$, there is $t_{v_{o_k}}=t_{o_k}$. Thus, the Observation Infection Time Order in ${{\textbf {TO}}}_{\mathcal {O}}$ is consistent with the Measuring Infection Time Order in ${{\textbf {TO}}}_{\mathcal {T}_{{\text {DIS}},s}}$. According to the Definition of correlation coefficient $\tau$ (Appendix A), $\tau \left( {{\textbf {TO}}}_{\mathcal {O}},{{\textbf {TO}}}_{\mathcal {T}_{{\text {DIS}},s}}\right) =1$. Property 2 is proved. $\square$

## C. Supplemental material

The interquartile range (IQR) of error hop of Gauss, GSSI, TRBS, SNF, OSBFS and OSDIS algorithms on the real networks is shown in Figs. 22, 23, 24, 25, 26, 27, 28.
